# Comparison of virtual to true unenhanced abdominal computed tomography images acquired using rapid kV-switching dual energy imaging

**DOI:** 10.1371/journal.pone.0238582

**Published:** 2020-09-23

**Authors:** D. Olivia Popnoe, Chaan S. Ng, Shouhao Zhou, Harmeet Kaur, Hyunseon C. Kang, Evelyne M. Loyer, S. Cheenu Kappadath, A. Kyle Jones

**Affiliations:** 1 MD Anderson Cancer Center UT Health Graduate School of Biomedical Sciences, Houston, Texas, United States of America; 2 Department of Diagnostic Radiology, The University of Texas MD Anderson Cancer Center, Houston, Texas, United States of America; 3 Department of Biostatistics, The University of Texas MD Anderson Cancer Center, Houston, Texas, United States of America; 4 Department of Imaging Physics, The University of Texas MD Anderson Cancer Center, Houston, Texas, United States of America; Yonsei University, South Korea, REPUBLIC OF KOREA

## Abstract

**Objective:**

To compare “virtual” unenhanced (VUE) computed tomography (CT) images, reconstructed from rapid kVp-switching dual-energy computed tomography (DECT), to “true” unenhanced CT images (TUE), in clinical abdominal imaging. The ability to replace TUE with VUE images would have many clinical and operational advantages.

**Methods:**

VUE and TUE images of 60 DECT datasets acquired for standard-of-care CT of pancreatic cancer were retrospectively reviewed and compared, both quantitatively and qualitatively. Comparisons included quantitative evaluation of CT numbers (Hounsfield Units, HU) measured in 8 different tissues, and 6 qualitative image characteristics relevant to abdominal imaging, rated by 3 experienced radiologists. The observed quantitative and qualitative VUE and TUE differences were compared against boundaries of clinically relevant equivalent thresholds to assess their equivalency, using modified paired t-tests and Bayesian hierarchical modeling.

**Results:**

Quantitatively, in tissues containing high concentrations of calcium or iodine, CT numbers measured in VUE images were significantly different from those in TUE images. CT numbers in VUE images were significantly lower than TUE images when calcium was present (e.g. in the spine, 73.1 HU lower, p < 0.0001); and significantly higher when iodine was present (e.g. in renal cortex, 12.9 HU higher, p < 0.0001). Qualitatively, VUE image ratings showed significantly inferior depiction of liver parenchyma compared to TUE images, and significantly more cortico-medullary differentiation in the kidney.

**Conclusions:**

Significant differences in VUE images compared to TUE images may limit their application and ability to replace TUE images in diagnostic abdominal CT imaging.

## Introduction

Multiphasic computed tomography (CT) examinations are a common and critically important non-invasive imaging technique for diagnosis and classification of abdominal lesions [1–3]. These examinations include a “pre-contrast” (referred to herein as unenhanced) phase followed by the intravenous administration of an iodinated contrast agent and one or more post-contrast imaging phases. Unenhanced images are used for both qualitative and quantitative interpretation. It has been proposed that “virtual” unenhanced images reconstructed from dual-energy computed tomography (DECT) data might be used to replace the initial pre-contrast imaging phase during multiphasic CT examinations [4–8].

Potential advantages of eliminating the unenhanced imaging phase include reduced radiation dose, reduced examination time, and fewer registration errors due to patient motion. It has been reported that radiation dose reductions of up to 35% can be realized if the unenhanced imaging phase is eliminated [2, 5, 9] and that mis-registration artifacts between unenhanced and enhanced phases can be eliminated [10] when DECT is used. However, there have also been reports that clinically important differences between “virtual” unenhanced (VUE) and “true” unenhanced (TUE) images may exist [4, 5, 7, 9, 11]. These differences may be expected when considering the physics of DECT, calibration of DECT scanners, and the computational methods used for reconstruction of VUE images.

Currently, there are five commercially-available technologies used to acquire and reconstruct DECT data, including rapid kVp-switching [11–13] dual source CT [5–7, 9, 10], single source sequential scanning, split filter CT [14], and detection based DECT using a dual layer detector [15].

The purpose of this retrospective study was to compare VUE images, reconstructed from DECT datasets acquired via rapid kVp-switching, with contemporaneously acquired TUE images in the setting of abdominal imaging.

## Methods and materials

This was a retrospective study which was approved by the Institutional Review Board of the University of Texas M.D. Anderson Cancer Center, who granted a waiver of informed consent. This study included both a quantitative comparison of CT numbers (Hounsfield Units, HU) measured in virtual unenhanced (VUE) and true unenhanced (TUE) CT images, and a qualitative review of specific image characteristics by experienced abdominal radiologists.

DECT datasets acquired as part of routine standard-of-care clinical practice for the evaluation of pancreatic cancer were used in this study. The CT images had been obtained on a CT scanner with rapid kVp-switching capability (Discovery HD750 CT scanner, General Electric Healthcare, Waukesha, WI) [16, 17]. A total of 60 consecutive DECT studies in patients with pancreatic cancer were evaluated. All studies included pre-contrast (unenhanced) and multiple post-contrast phases.

The unenhanced phase was acquired at 120 kVp using in-plane and longitudinal tube current modulation (TCM) at a pitch of 0.984, rotation time of 0.8 s, and a 64 x 0.625 detector configuration. The unenhanced scan started at the dome of the liver and continued through the iliac crests. The Noise Index (NI) for the unenhanced phase was 18.0 or 20.7, depending on patient size (display field of view [DFOV] up to 40 cm and 42 cm or greater, respectively), with a median reported CTDI_vol_ of 9.30 mGy (range, 9.06 mGy to 18.2 mGy).

Three post-contrast phase scans were acquired, consisting of late arterial, portal venous, and delayed phases. VUE images were generated from the late arterial phase (“pancreatic parenchymal”), which was acquired using rapid kVp-switching DECT (80/140 kVp) with Gemstone Spectral Imaging (GSI) preset GSI-10 or GSI-5 at a pitch of 0.984 and rotation time of 0.8 s. An injection of 125 cc of iodinated contrast (Optiray 350, Mallinckrodt, Inc., Hazelwood, MO) was performed at a rate of 4 mL/s with a scan delay of 20 s after 100 HU enhancement was reached in the aorta at the level of the celiac artery. The scan range covered from the dome of the liver to just inferior to the iliac crests. The late arterial phase corresponds to the cortico-medullary phase of renal enhancement.

DECT data was processed using version 2.0 of the Gemstone Spectral Imaging (GSI) Volume Viewer (GE Healthcare, Waukesha, WI). Included with this software was the Material Suppressed Iodine (MSI) feature, which was used to reconstruct Virtual Unenhanced (VUE) images from the DECT dataset. The MSI feature uses a novel multi-material decomposition algorithm that attempts to replace the calculated volume of iodine contrast in a voxel with an equivalent volume of blood to generate VUE images [18, 19].

Iterative reconstruction (Adaptive Statistical Iterative Reconstruction [ASiR], GE Healthcare) had been applied to the routine clinical TUE images using a 60% ASiR level but not to the DECT datasets. TUE images were reconstructed using the SOFT kernel and VUE images using the STANDARD kernel. Both TUE and VUE images were reconstructed at an image thickness of 3.75 mm and interval of 2.5 mm and the display field of view (DFOV) for all VUE images were matched to the TUE images for the same patient.

### Quantitative evaluation of CT number

**Fig 1:**
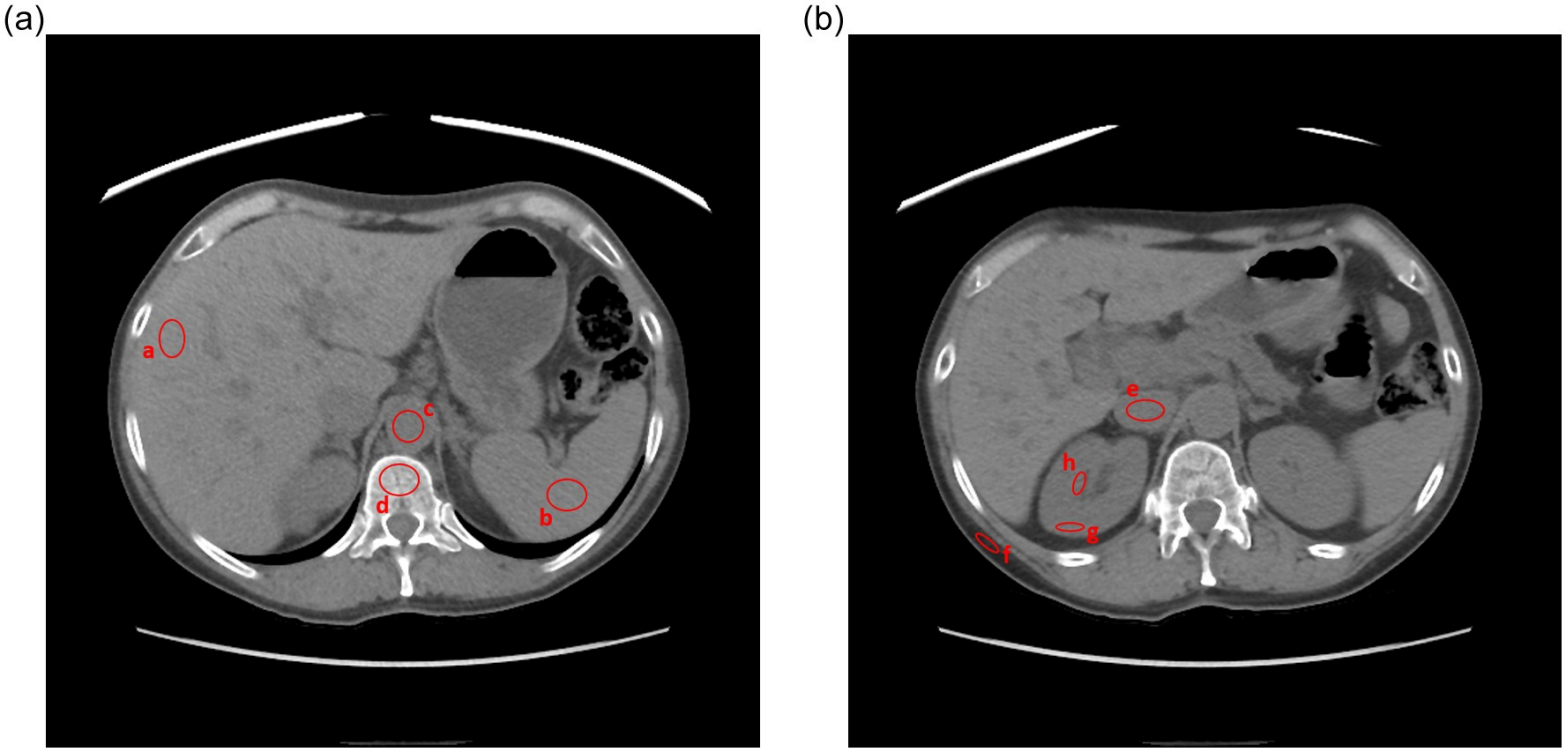
Typical region of interest (ROI) placement for the quantitative analysis. a) liver; b) spleen; c) aorta; d) spine; e) inferior vena cava (IVC); f) subcutaneous fat; g) renal cortex; h) renal medulla.

CT numbers measured in human tissues were compared between TUE images and VUE images in the series of DECT studies acquired using the aforementioned imaging protocol. TUE and VUE images were registered using the “Integrated Registration” feature of the GSI Volume Viewer and a single elliptical region of interest (ROI) was placed in each tissue of interest in the registered datasets by a single observer (D.O.P) to return the mean CT number in the same location on both the TUE and VUE images. The size of the ROI ranged from 30 mm^2^ to 700 mm^2^ depending on the tissue being measured. Comparisons were made in the right lobe of the liver, abdominal aorta above the renal arteries, inferior vena cava (IVC) above the level of the renal veins, spleen, spine (at the L1 level), renal medulla, renal cortex, and subcutaneous fat at the level of the kidneys (Fig 1). Care was taken to avoid major vascular structures and lesions when obtaining CT number measurements in the organs, i.e., the liver, spleen, kidneys.

### Qualitative image evaluation

The same series of TUE and VUE CT images were presented to three subspecialty trained abdominal radiologists with post-fellowship clinical experience of 4, 10, and 25 years (H.C.K, H.K, E.M.L). A random subset of 15 studies from the cohort of 60 studies, which all included both TUE and VUE images, was presented twice to each observer, blindly and randomly, without their prior knowledge, to assess intra-observer variability as an input to the Bayesian hierarchical model used for data analysis. Therefore, a total of 150 image sets were presented to each radiologist, and images were presented during multiple sessions to minimize radiologist fatigue. The 150 image sets consisted of 60 TUE, 60 VUE, 15 randomly selected repeated TUE and 15 randomly selected repeat VUE image sets. TUE and VUE image series were presented one at a time in random order on a clinical PACS workstation. Each radiologist was asked to review and rate the following image characteristics: visualization of major vessels (considering hepatic, portal, and renal veins in particular); sharpness of the pancreatic contour; visualization of calcifications; depiction of liver parenchyma; visualization of adrenal glands; and cortico-medullary differentiation. All characteristics, except cortico-medullary differentiation, were rated on a 5-point Likert scale (1 = Very poor, 2 = Poor, 3 = Adequate, 4 = Good, 5 = Very good). Cortico-medullary differentiation was evaluated on a 3-point Likert scale (1 = No differentiation, 2 = Slight differentiation, 3 = Extreme differentiation).

Radiologists were allowed to manipulate images, including adjusting window width and level, as they would for routine clinical interpretation. Prior to each review session, radiologists were provided instructions and example images not included in the study that demonstrated the baseline for each characteristic to be rated.

### Statistical methods

For both the quantitative and qualitative evaluations, the goals of the statistical analyses were to determine if the observed differences between VUE and TUE images were within, or outside, acceptable *clinical* tolerances (or “equivalence thresholds”). The equivalence thresholds were specified as the range of: a) HU differences, in the case of the quantitative evaluation, and b) Likert scores, in the case of the qualitative evaluation, within which the observed differences in VUE and TUE would be considered equivalent. The data analyses were conducted using statistical software R v3.4.3 <https://www.r-project.org> with packages rjags_v4-6 and coda_v0.19–1.

For the quantitative evaluation, the equivalence threshold was determined with reference to the CT scanner manufacturer’s expectations of CT number measurement tolerance. The technical reference manual [20] for the GE Discovery HD 750 CT scanner specified a quality control tolerance of 0 ± 6 HU at 120 kVp for the CT number of water, which is equivalent to a difference in linear attenuation coefficient of ± 0.6%. For each tissue, the average CT number measured on the TUE images was converted to a linear attenuation coefficient, and a ± 0.6% range was calculated about each number. The average of the upper and lower bounds was calculated, converted back to CT number, and used as the threshold of a clinically relevant difference for each tissue.

For each tissue in the quantitative evaluation, we summarized the average CT numbers of VUE and TUE images. The difference (Δ) between measured CT numbers in VUE and TUE images was calculated for each patient and a 95% confidence interval (CI) for the mean difference was calculated and compared to the above equivalence threshold difference. A p-value was calculated, based on a modified one-tailed paired t-test against the threshold, to examine the null hypothesis, which was that the absolute difference between VUE and TUE images was not greater than the calculated threshold difference. If a p-value was <0.05, or equivalently, if the 95% CI was either completely *above* the upper threshold or completely *below* the lower threshold, the difference was considered significant and the null hypothesis was rejected.

Data from the qualitative evaluation was analyzed using Bayesian hierarchical modeling that incorporated random effects for factors including image source (study), image characteristic, observer (radiologist), repeated evaluation, and the nested effects among those factors to justify intra- and inter-observer variability and variance decomposition [21]. JAGS v4.3.0 <http://mcmc-jags.sourceforge.net> was applied for the simulation and the inference was drawn based on 10,000 valid posterior samples after convergence. Deviance Information Criterion was applied for model selection [22].

In consideration of the discrete nature of the Likert rating scale, the equivalence threshold for the qualitative evaluation was determined with reference to the minimum discernable difference between two adjacent Likert score, i.e. ±0.5. For each imaging feature in the qualitative evaluation, the posterior median, standard deviation, and 95% credible interval (CrI) of the difference of Likert scores between VUE and TUE images (β) was estimated and compared to the above equivalence threshold of ±0.5. If the 95% CrI of the model did not include ±0.5 of the Likert score, i.e., lay outside the equivalence threshold, then VUE was considered statistically different from TUE. Given the ordinal definitions assigned to the Likert scores, a conservative difference of *-0*.*5 or less* (<-0.5) between VUE and TUE image ratings (i.e. VUE minus TUE) was considered to indicate clinical *inferiority* of VUE images compared to TUE images. Of note, in the case of cortico-medullary differentiation, a *positive* difference in scores indicated more pronounced differentiation.

## Results

A total of 60 consecutive DECT studies of patients with pancreatic cancer (28 female and 32 male patients) were evaluated both quantitatively and qualitatively. The median body mass index (BMI) of scanned patients was 26.1 kg/m^2^ (range, 15.0 kg/m^2^ to 44.7 kg/m^2^).

**Fig 2:**
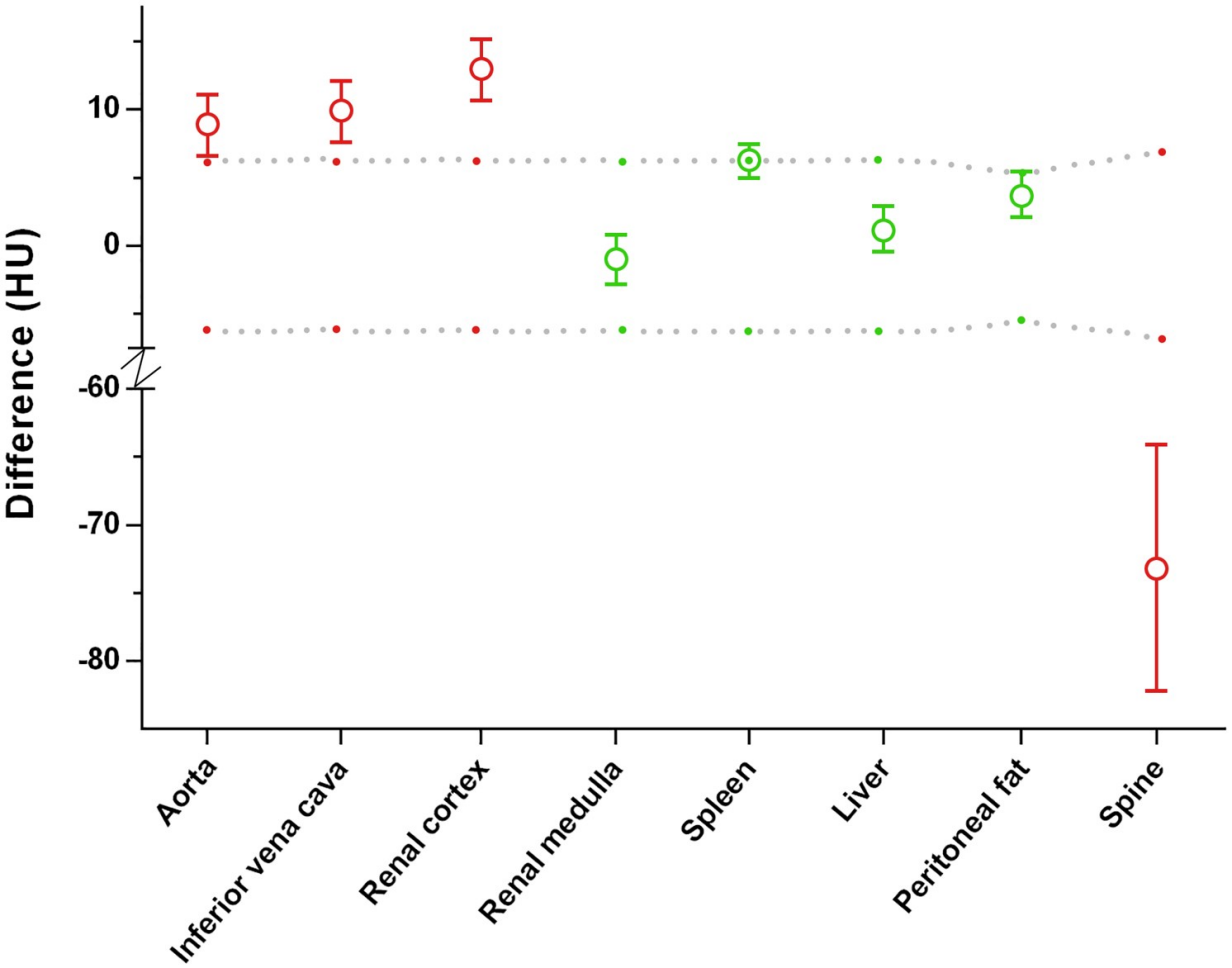
Quantitative differences between VUE and TUE images, based on measured CT numbers. y-axis represents differences in CT number (HU) for VUE minus TUE. Red and green symbols represent the measured differences and 95% CI in CT numbers between VUE and TUE images. Gray dots reflect the calculated boundary of clinical equivalence between VUE and TUE (see text for explanation). Green symbols indicate that the measured difference between VUE and TUE lies within the calculated threshold range of equivalence. Red symbols indicate that the measured difference between VUE and TUE images is significantly greater than (i.e. outside) the calculated threshold difference of equivalence.

Table 1 summarizes the results of the quantitative evaluation by comparing the CT numbers measured in tissues in registered TUE and VUE images. In tissues with substantial calcium content (e.g. spine), CT numbers in VUE images were significantly *lower* than TUE images (Δ = -73.1 HU, 95% CI (-63.9, -82.4); p < 0.0001). In tissues with substantial iodine contrast content in the contrast-enhanced phase (e.g. aorta, IVC and renal cortex), the CT numbers measured in VUE images were significantly *greater* than those measured in TUE images, with the largest difference observed in renal cortex (Δ = 13.0 HU higher, 95% CI (10.7, 15.3); p < 0.0001). In tissues without substantial calcium or iodine content in the contrast enhanced images (e.g. liver, spleen, renal medulla, and subcutaneous fat), the mean differences in CT numbers measured between TUE and VUE were not greater than the threshold differences. The mean differences between TUE and VUE were borderline significant for the spleen (Table 1, Fig 2). In general, the direction of the difference depended on whether calcium or iodine was present in the tissue during the contrast enhanced phase.

**Table 1:** Results of the quantitative image evaluation, based on CT number (HU).

Tissue	TUE mean measured CT number (HU)	VUE mean measured CT number (HU)	Calculated equivalence threshold (HU)	95% CI for measured difference, (Δ = VUE-TUE, HU)	P-value	Significance
Aorta	35.9	44.8	± 6.2	**(6.5, 11.2)**	**0.036**	***
IVC	29.4	39.2	± 6.2	**(7.4, 12.3)**	**0.002**	***
Renal Cortex	27.5	40.4	± 6.2	**(10.7, 15.3)**	**<0.0001**	***
Renal Medulla	29.1	28.1	± 6.2	(-2.9, 0.9)	1.0	ns
Liver	51.2	52.3	± 6.3	(-0.9, 3.1)	1.0	ns
Spleen	41.4	47.6	± 6.3	(5.0, 7.6)	0.49	ns
Fat	-99.2	-95.6	± 5.4	(1.7, 5.2)	0.99	ns
Spine	155.3	82.2	± 6.9	**(-82.4, -63.9)**	**<0.0001**	***

***Indicates that VUE images are significantly different compared to TUE images.

ns = non-significant difference between VUE and TUE images, i.e. the 95% CI for difference between VUE and TUE lies within (encompasses) or intersects with the region between the equivalence thresholds

**Fig 3:**
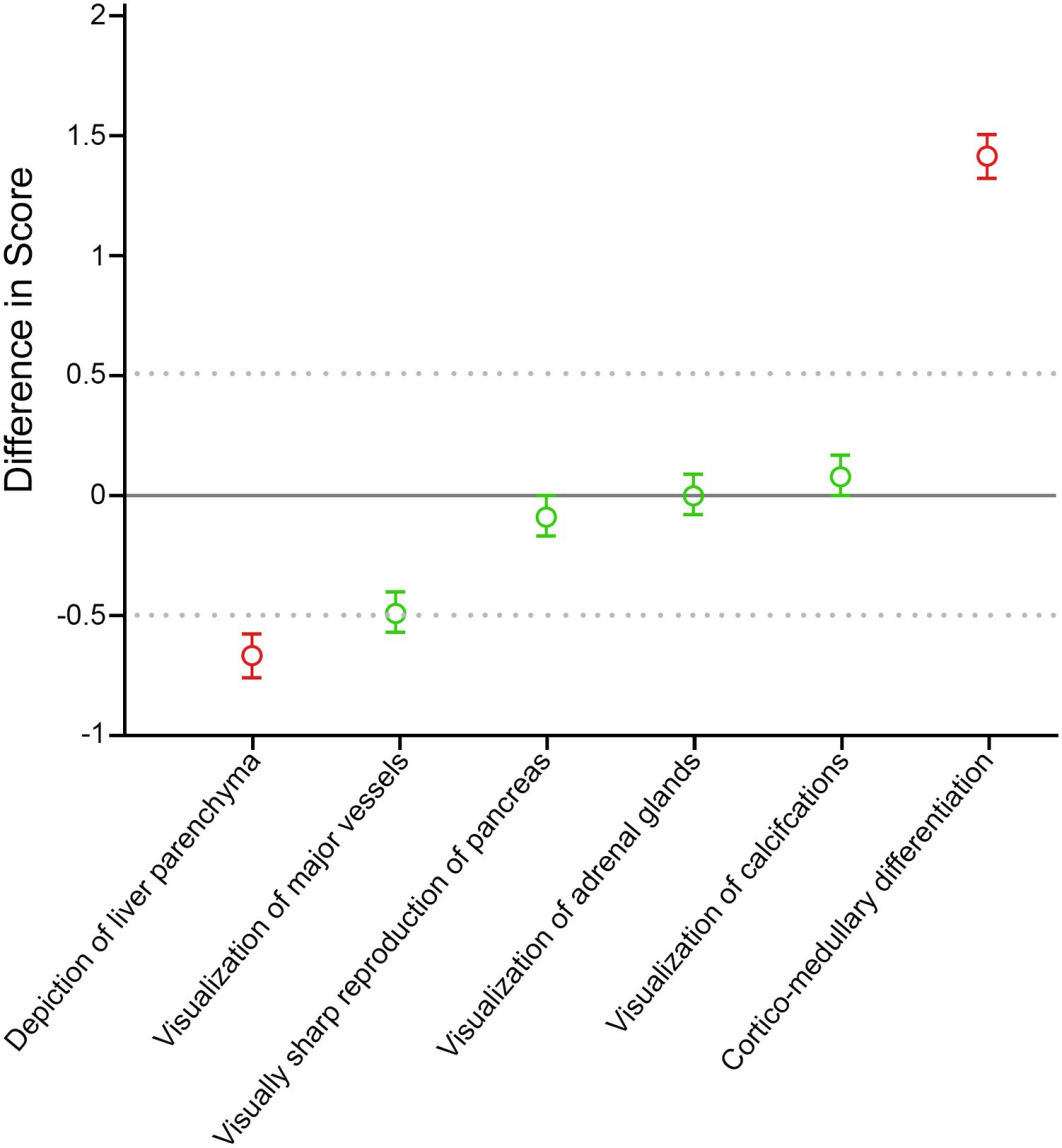
Qualitative differences between VUE and TUE images, based on Likert scores of radiologists. y-axis represents differences in Likert scores for VUE minus TUE. Red and green symbols represent the mean differences (β) and model 95% credible interval (CrI) in radiologists’ Likert scores between VUE and TUE images (see text for explanation). Gray dots reflect the boundary of clinical equivalence between VUE and TUE (±0.5 difference in Likert scores). Green symbols indicate that the measured difference between VUE and TUE lies within the calculated threshold range of equivalence. Red symbols indicate that the measured difference between VUE and TUE images is significantly greater than (i.e. outside) the calculated threshold difference of equivalence. Negative differences indicate inferiority of VUE compared to TUE; except for cortico-medullary differentiation, where a positive difference connotes greater differentiation.

Table 2 summarizes the results from the qualitative comparison of TUE and VUE images based on Bayesian hierarchical modeling. Among the qualitative imaging features assessed, VUE images were rated *inferior* to TUE images for depiction of liver parenchyma (β = -0.68, 95% CrI (-0.79, -0.58)); and VUE images were rated as showing significantly more cortico-medullary differentiation than TUE images (β = 1.38, 95% CrI (1.30, 1.46)). Visualization of major vessels had a mean model difference that suggested borderline inferiority of VUE compared to TUE (β = -0.51), but its 95% credible interval contained the range ±0.50, and thus was not formally statistically different (Table 2, Fig 3).

**Table 2:** Results of the qualitative image rating evaluation by radiologists, based on Likert scores.

Imaging Characteristic	Model difference in Likert score (β)	Standard deviation of model difference	95% Credible Interval (CrI) for the observed difference between VUE and TUE	Clinical significance
Depiction of liver parenchyma	-0.68	0.05	**(-0.79, -0.58)**	***
Visualization of major vessels (hepatic, portal, and renal veins in particular)	-0.51	0.06	(-0.62, -0.41)	ns
Visually sharp reproduction of the pancreatic contour	-0.09	0.05	(-0.20, 0.004)	ns
Visualization of adrenal glands	-0.01	0.06	(-0.10, 0.08)	ns
Visualization of calcifications	0.13	0.05	(0.03, 0.22)	ns
Cortico-medullary differentiation	1.38	0.04	**(1.3, 1.46)**	***

***indicates significant difference between VUE and TUE images, i.e. the 95% credible interval lies outside the equivalence range of ±0.5 ns = indicates no significant difference between VUE and TUE images, i.e. the 95% credible interval lies within (encompasses) or intersects with the region between equivalence range of Likert score ±0.5

The specific design of the evaluation of imaging features and the Bayesian hierarchical model allowed discrimination of the inter- and intra-observer components of overall variation in the qualitative study. The results showed that inter-observer variation (β = 0.56, 95% CrI (0.51, 0.61)) was significantly larger than intra-observer variation (β = 0.35, 95% CrI (0.05, 0.43)).

## Discussion

Our results suggest that significant quantitative and qualitative differences exist between VUE and TUE images. Depending on the tissue and feature examined, these differences have the potential to substantially impact the accurate interpretation of clinical radiologic images.

**Fig 4:**
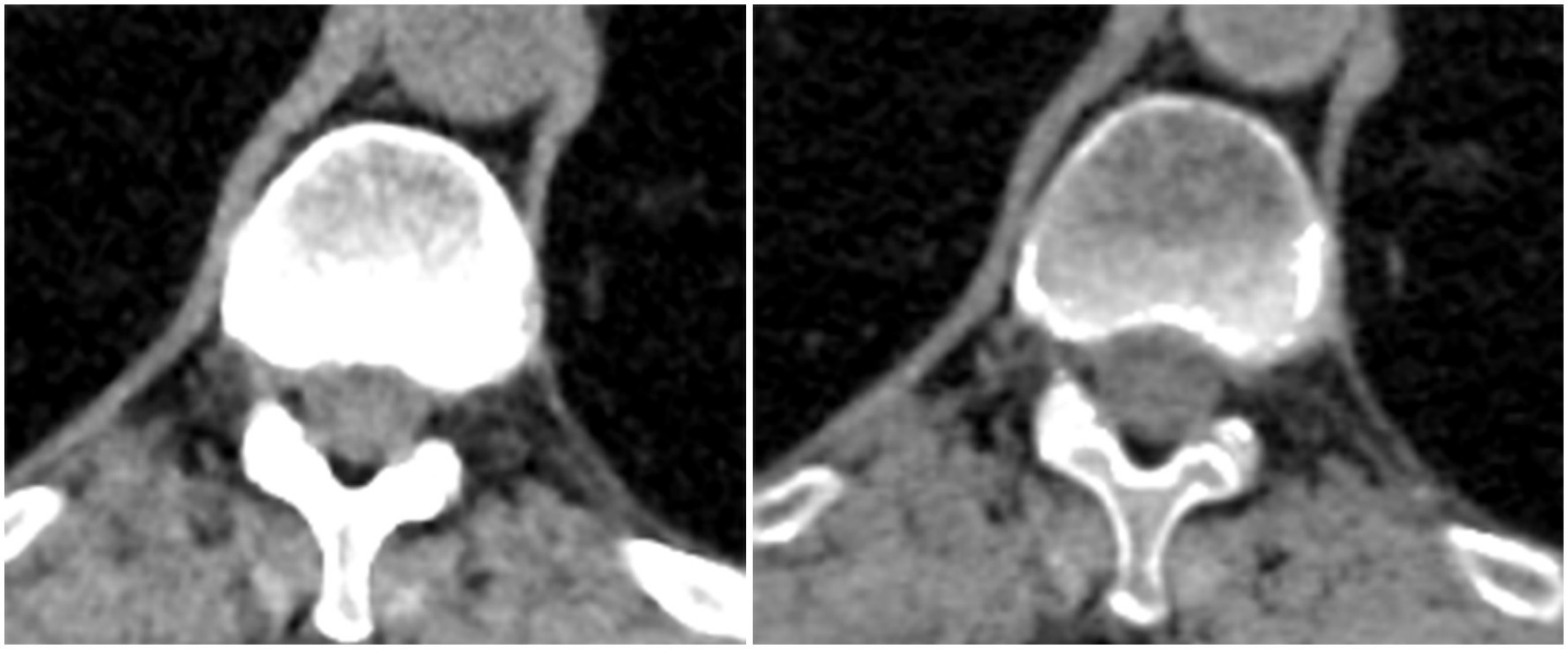
Example of calcium subtraction artifact. Same location in true unenhanced (TUE) image (left) and virtual unenhanced (VUE) image (right). Images are displayed using the same CT window width and window level.

**Fig 5:**
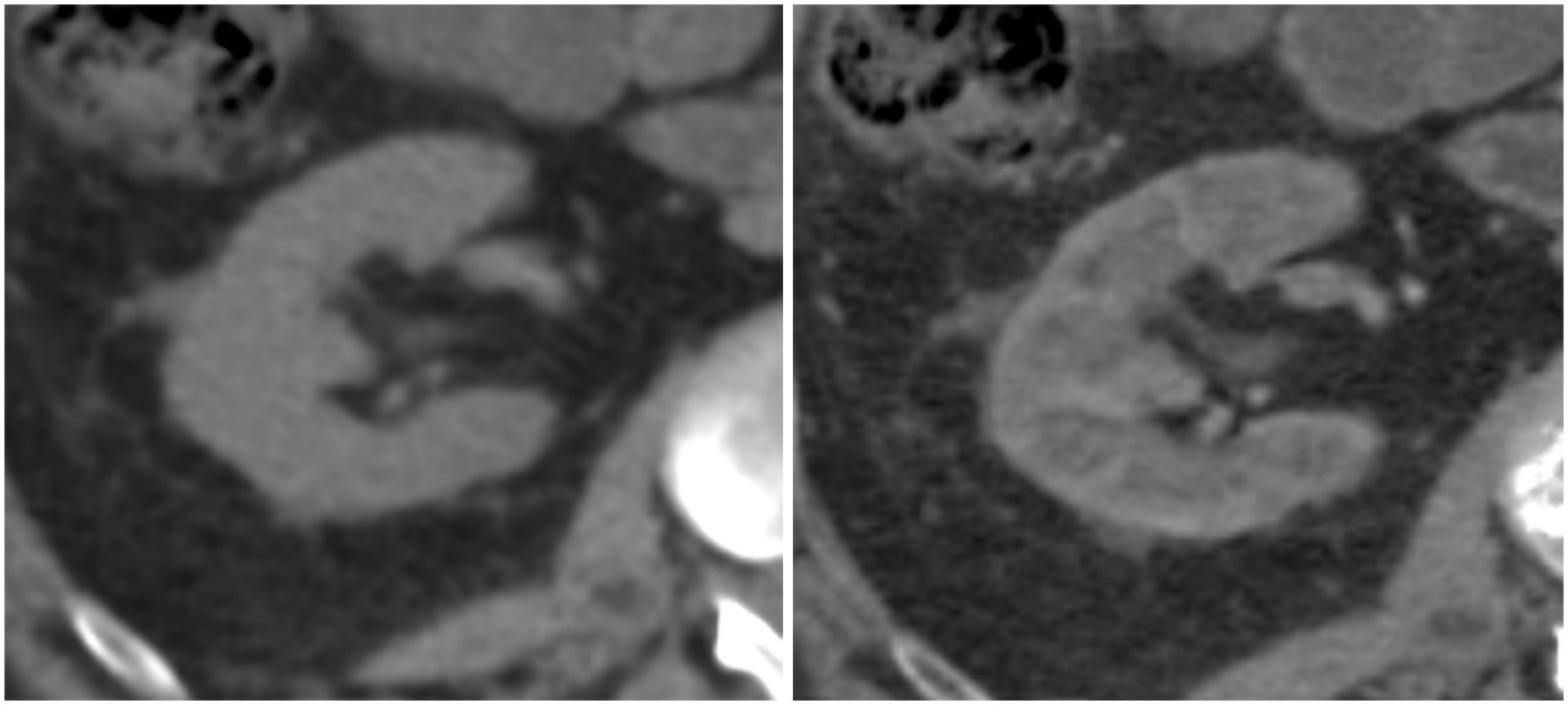
No cortico-medullary differentiation is visible on true unenhanced (TUE) images (left) while virtual unenhanced (VUE) images demonstrate cortico-medullary differentiation (right). Images are displayed using the same CT window width and window level.

Quantitatively, the amount of calcium or iodine present in a tissue on the contrast-enhanced phase of DECT images had significant impact, and indeed most strongly influenced the objective differences between VUE and TUE images (Fig 2). In bone, the partial removal of calcium in VUE images, referred to as the “subtraction artifact”, resulted in a CT number that was *significantly lower* than the CT number measured in TUE images (Fig 4). In tissues containing substantial iodine, such as the renal cortex, the incomplete removal of iodine resulted in VUE CT numbers that were *significantly higher* than TUE CT numbers (Fig 5). The observed differences between VUE and TUE in tissues followed the expected iodine content of different tissues during the late arterial phase of contrast enhancement: for example, renal cortex greater than renal medulla, and aorta and inferior vena cava greater than liver or spleen (Fig 2).

Based on radiologists’ qualitative ratings of imaging features, cortico-medullary differentiation (β = 1.38) and the depiction of liver parenchyma (mean model difference, β = -0.68) were significantly different between VUE and TUE images. Cortico-medullary differentiation is not normally appreciable in noncontrast images, and therefore was not observed on TUE images. VUE images, on the other hand, were reconstructed from the DECT dataset acquired during the cortico-medullary phase of renal enhancement, and incomplete removal of iodine from the avidly enhancing renal cortex resulted in visualization of cortico-medullary differentiation on VUE images (Fig 5). This is an inherent artifact of the MSI algorithm.

Depiction of liver parenchyma was found to be inferior on VUE images compared to TUE images. Detection and characterization of liver lesions would be negatively affected by misrepresents or distortions of the appearance of the background liver parenchyma. Smaller lesions could go unnoticed if margins were blurry. The borderline inferior rating observed for major vessels (β = -0.5), specifically hepatic and portal veins, as a surrogate for the margins of lesions, is consistent with the overall inferior rating of the depiction of liver parenchyma.

Previous studies have reported inconsistent results for CT numbers measured in virtual unenhanced images. A phantom study by Popnoe et al. found that measured enhancement was significantly lower when using VUE images compared to TUE images when any amount of iodine contrast was present [13]. However, accuracy for classifying lesions as “non-enhancing” (< 15 HU) or “enhancing” (> 15 HU) was negatively impacted only for enhancing lesions, as incomplete removal of iodine content tended to make CT numbers in VUE images higher than those in TUE images. Graser et al. studied renal mass protocol imaging using dual source DECT and reported no significant difference between CT numbers in TUE and VUE images for several tissues, however, they did not report where in the renal parenchyma ROI measurements were made [2]. Additionally, the study involved a three material decomposition algorithm used for the reconstruction of virtual unenhanced images from dual energy data as opposed to the two material decomposition method used in this work. In contrast, Sahni et al. reported significant differences in measured CT numbers between TUE and VUE images when using dual source DECT for patients undergoing CT urography [9], and Kawamoto et al. reported similar findings for patients undergoing CT urography using first generation dual source DECT but found no difference when a second generation dual source DECT with a tin filter added to the high energy beam was used [7]. Kaza et al. reported measured CT number differences of 5–9 HU between TUE and VUE images produced with rapid kVp-switching DECT for follow up after renal mass ablation, with no significant differences observed between the cortico-medullary and nephrographic phases [11]. Euler et al. reviewed patient thoraco-abdominal CT images acquired using single source DECT with a split filter and reported mean absolute differences between virtual and true non-contrast images ranging from 3.1–6.7 HU in visceral organs, vessels, and muscle [14]. They also reported that in both a phantom study and patient image review, larger relative differences were observed at lower iodine concentrations. While all of the aforementioned investigators found that, when different, CT numbers in VUE images were greater than those in TUE images, De Cecco et al. reported that for the pancreas, renal medulla, adrenal gland, and aorta (arterial phase) and for the portal vein, renal cortex, and retroperitoneal fat (portal venous phase), CT numbers measured in VUE images were *less than* those measured in TUE images [5]. Euler et al. evaluated specific aspects of image quality subjectively and found that while scores were slightly lower for DECT, there were no significant differences in subjective image evaluation for noise, contrast, artifacts, sharpness, and diagnostic confidence [14]. Observer-quantified image quality of VUE images in these studies has indicated that VUE images are generally of lower overall quality than TUE images but still acceptable, with no report of the performance of VUE for specific image characteristics [2, 4, 9–11]. Ananthakrishnan et al. studied VUE images created using detection-based spectral CT with a dual layer detector and found that among all tissues and organs evaluated, only subcutaneous fat had VUE CT numbers that were “not equivalent”, defined as a difference of greater than 10 HU, to those measured in TUE images [15].

We recognize and acknowledge other limitations in our study. The reviewing radiologists provided anecdotal feedback that the question related to visibility of calcifications was treated as a binary question, either present or not present, without consideration of the number of calcifications seen. Therefore, the mean model difference may not accurately reflect the true difference in the visibility of calcifications between TUE and VUE images. Calcium subtraction artifacts in virtual unenhanced images have been reported previously [2, 4] and are likely unavoidable when calcium is not considered as one of the basis pair materials (in this case iodine and water) in the material decomposition algorithm.

Our study included qualitative evaluation and scoring of images, which inevitably has some degree of subjectivity. However, we mitigated these effects by incorporating multiple observers and the observers were subspecialty and fellowship trained. Furthermore, our study design, which included blinded repetition of a subset of studies, and the use of Bayesian hierarchical modeling allowed us to assess inter- and intra-observer variation. This showed that within-observer variation was smaller than between-observers, which is concordant with expectations.

**Fig 6:**
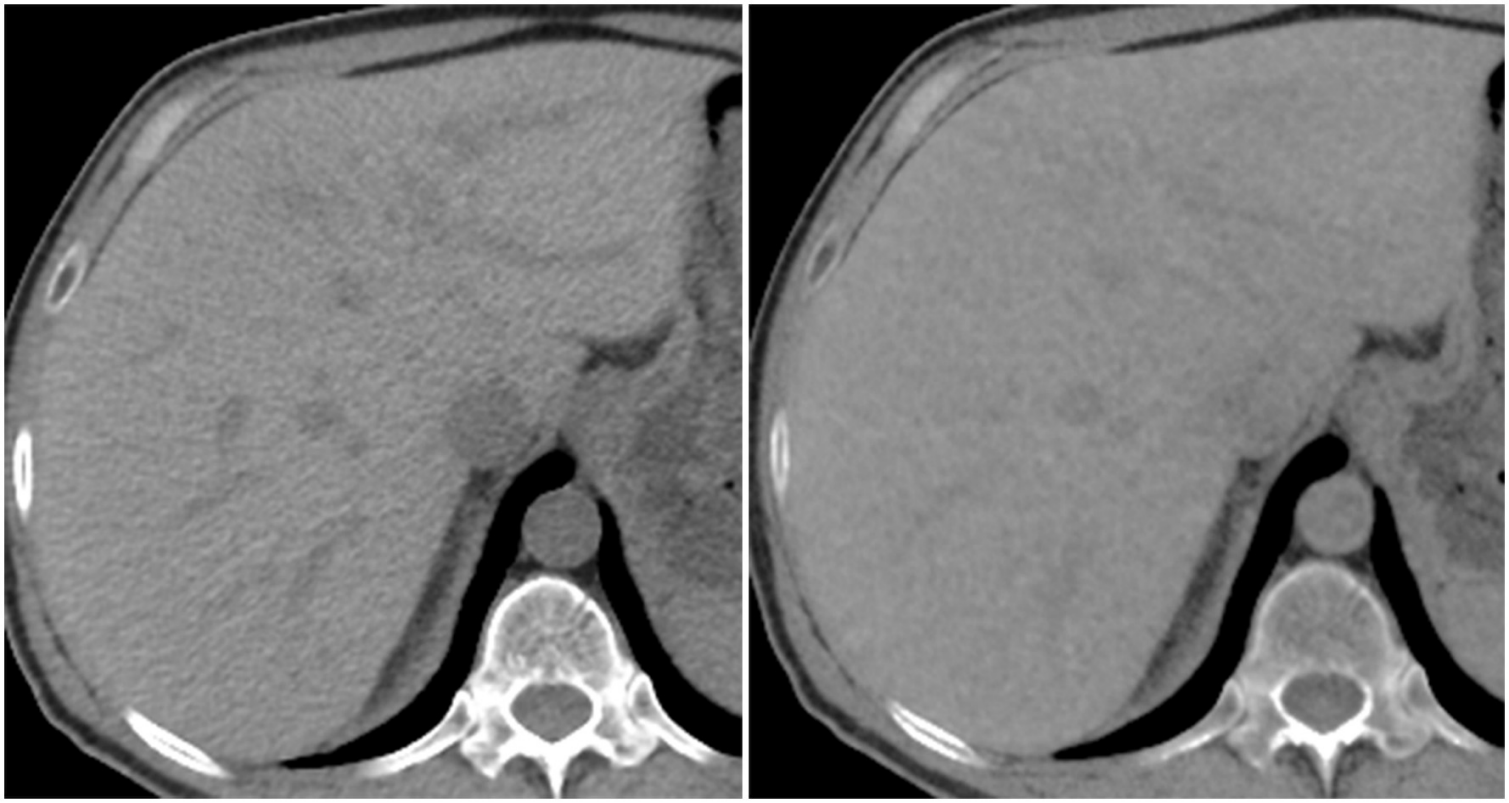
Appearance of liver parenchyma and venous structures on true unenhanced (TUE) image (left) and virtual unenhanced (VUE) image (right). Images are displayed using the same CT window width and window level.

The TUE and VUE images evaluated in this study had been reconstructed with different kernels (SOFT vs. STANDARD) and with different levels of iterative reconstruction. These had been reconstructed as such at the time of acquisition and for clinical interpretation, and unfortunately, the raw CT data was no longer available to allow us the opportunity to harmonize these aspects. This may have affected the qualitative evaluations in our study, particularly in the depiction of liver parenchyma and potentially for visualization of major vessels. Indeed, image noise was noticeably different between the VUE and TUE images (Fig 6), however, given that neither kernel was a “sharp” kernel, no difference in CT number would be expected when using either kernel [23] This was a potential confounding factor for the evaluation of liver parenchyma, and a limitation of this study. Interpretation of the mean model difference for calcification visibility was confounded by the binary manner in which the question was approached by the radiologists, however, it is clear from Fig 2 that there was a difference in calcification visibility between VUE and TUE images. It is also possible that some observer bias could have existed, as the clear presence of cortico-medullary differentiation in all VUE images meant that observers were essentially unblinded as to which images were TUE and VUE.

However, neither CT number nor cortico-medullary differentiation would be affected by the difference in kernel or iterative reconstruction between VUE and TUE images. As a result, it is clear that there are differences, both quantitative and qualitative, between VUE and TUE images. While most perceptual differences are obviously artifactual, the potential for removal of small calcifications is concerning. Of more concern is the potential impact on quantification if VUE images are used as the baseline for calculation of enhancement, as the results of this study indicate that CT numbers measured in tissues containing substantial amounts of iodine contrast are significantly higher in VUE images compared to TUE images. Further study is required to investigate this concern, especially considering that it has been reported that single-phase DECT allows for characterization of renal masses as benign or malignant [10] even as questions remain about the use of DECT to accurately characterize hyper-attenuating renal masses [10] and equivocally enhancing renal masses [12]. The concern extends to characterization of lesions in other organs, including the liver.

In conclusion, although VUE and TUE images showed comparability across several tissues and imaging characteristics, significant quantitative and qualitative differences exist with respect to some clinically important areas. The latter include quantification of CT numbers in iodine- and calcium- containing tissues, and qualitative evaluation of liver parenchyma and renal tissue. Overall, this work does not support the replacement of true unenhanced with virtual unenhanced images derived from rapid kVp-switching, at least with the specific DECT scanner and associated software investigated herein, for clinical diagnostic CT in the abdomen. Further investigation on the impact of the observed differences on the quantitative use of VUE images, particularly in regards to evaluation of enhancing masses, is needed.

## Supporting information

S1 Dataset (XLSX)

## References

[1] Bosniak MA. Diagnosis and management of patients with complicated cystic lesions of the kidney. AJR Am J Roentgenol. 1997; 169(3):819–21. Epub 1997/09/01. 10.2214/ajr.169.3.9275903 . 9275903

[2] Hennedige T, Venkatesh SK. Imaging of hepatocellular carcinoma: diagnosis, staging and treatment monitoring. Cancer Imaging. 2012; 12:530–47. Epub 2013/02/13. 10.1102/1470-7330.2012.0044 . 23400006 PMC3666429

[3] Kim SK, Lim JH, Lee WJ, Kim SH, Choi D, Lee SJ, et al Detection of hepatocellular carcinoma: comparison of dynamic three-phase computed tomography images and four-phase computed tomography images using multidetector row helical computed tomography. J Comput Assist Tomogr. 2002; 26(5):691–8. Epub 2002/11/20. 10.1097/00004728-200209000-00005 . 12439300

[4] Ascenti G, Mazziotti S, Mileto A, Racchiusa S, Donato R, Settineri N, et al Dual-source dual-energy CT evaluation of complex cystic renal masses. AJR Am J Roentgenol. 2012; 199(5):1026–34. Epub 2012/10/26. 10.2214/AJR.11.7711 . 23096175

[5] De Cecco CN, Muscogiuri G, Schoepf UJ, Caruso D, Wichmann JL, Cannao PM, et al Virtual unenhanced imaging of the liver with third-generation dual-source dual-energy CT and advanced modeled iterative reconstruction. Eur J Radiol. 2016; 85(7):1257–64. Epub 2016/05/29. 10.1016/j.ejrad.2016.04.012 . 27235872

[6] Graser A, Johnson TR, Hecht EM, Becker CR, Leidecker C, Staehler M, et al Dual-energy CT in patients suspected of having renal masses: can virtual nonenhanced images replace true nonenhanced images? Radiology. 2009; 252(2):433–40. Epub 2009/06/03. 10.1148/radiol.2522080557 . 19487466

[7] Kawamoto S, Zhou X, Leidecker C, Fung G, Tsui B, Fishman K. Virtual noncontrast renal imaging using dual-energy CT: evaluation of CT numbers of renal parenchyma and renal masses. Imaging in Medicine. 2011; 3(5):501–11.

[8] Mileto A, Nelson RC, Paulson EK, Marin D. Dual-Energy MDCT for imaging the renal mass. AJR Am J Roentgenol. 2015; 204(6):W640–7. Epub 2015/03/03. 10.2214/AJR.14.14094 . 25730444

[9] Sahni VA, Shinagare AB, Silverman SG. Virtual unenhanced CT images acquired from dual-energy CT urography: accuracy of attenuation values and variation with contrast material phase. Clin Radiol. 2013; 68(3):264–71. Epub 2012/09/15. 10.1016/j.crad.2012.08.004 . 22974566

[10] Graser A, Becker CR, Staehler M, Clevert DA, Macari M, Arndt N, et al Single-phase dual-energy CT allows for characterization of renal masses as benign or malignant. Invest Radiol. 2010; 45(7):399–405. Epub 2010/05/26. 10.1097/RLI.0b013e3181e33189 . 20498609

[11] Kaza RK, Raff EA, Davenport MS, Khalatbari S. Variability of CT attenuation measurements in virtual unenhanced images generated using multimaterial decomposition from fast kilovoltage-switching dual-energy CT. Acad Radiol. 2017; 24(3):365–72. Epub 2016/10/23. 10.1016/j.acra.2016.09.002 . 27769822

[12] Kaza RK, Caoili EM, Cohan RH, Platt JF. Distinguishing enhancing from nonenhancing renal lesions with fast kilovoltage-switching dual-energy CT. AJR Am J Roentgenol. 2011; 197(6):1375–81. Epub 2011/11/24. 10.2214/AJR.11.6812 22109292

[13] Popnoe OD, Ng CS, Zhou S, Kappadath CS, Pan T, Kyle Jones A. Comparison of enhancement quantification from virtual unenhanced images to true unenhanced images in multiphase renal Dual-Energy computed tomography: A phantom study. J Appl Clin Med Phys. 2019; 20(8):171–9. Epub 2019/08/20. 10.1002/acm2.12685 . 31423728 PMC6698809

[14] Euler A, Parakh A, Falkowski AL, Manneck S, Dashti D, Krauss B, et al Initial results of a single-source dual-energy computed tomography technique using a split-filter: Assessment of image quality, radiation dose, and accuracy of dual-energy applications in an in vitro and in vivo study. Invest Radiol. 2016; 51(8):491–8. Epub 2016/02/20. 10.1097/RLI.0000000000000257 . 26895193

[15] Ananthakrishnan L, Rajiah P, Ahn R, Rassouli N, Xi Y, Soesbe TC, et al Spectral detector CT-derived virtual non-contrast images: comparison of attenuation values with unenhanced CT. Abdom Radiol (NY). 2017; 42(3):702–9. Epub 2017/01/14. 10.1007/s00261-016-1036-9 . 28084546

[16] Chandra N, Langan DA. Gemstone Detector: Dual energy imaging via fast kVp switching Dual Energy CT in Clinical Practice. New York, NY: Springer, 2011:35–41. Epub 22 September 2010. 10.1007/174_2010_35.

[17] Xu D, Langan DA, Wu X, Pack JD, Benson TM, Tkaczky JE, et al., editors. Dual energy CT via fast kVp switching spectrum estimation. Proc SPIE 7258, Medical Imaging 2009: Physics of Medical Imaging; 13 March 2009.

[18] Maddah M, Mendonça PR, Bhotika R, editors. Physically meaningful virtual unenhanced image reconstruction from dual-energy CT. 2010 IEEE International Symposium on Biomedical Imaging: From Nano to Macro; 2010.

[19] Mendonça PR, Bhotika R, Maddah M, Thomsen B, Dutta S, Licato P, et al., editors. Multi-material decomposition of spectral CT images. SPIE 7622, Medical Imaging 2010: Physics of Medical Imaging 2010.

[20] GE Healthcare. DiscoveryTM CT750 HD Technical Reference Manual Rev. 8. 5317222-1EN 2011.

[21] Gelman A, Carlin J, Stern H, Dunson D, Vehtari A, Rubin D. Bayesian data analysis. Third edition ed. Raton Boca: Chapman and Hall/CRC Press; 2013.

[22] Spiegelhalter D, Best N, Carlin B, Van Der Linde A. Bayesian measures of model complexity and fit. Journal of the royal statistical society: Series b (statistical methodology). 2002; 64(6):583–639.

[23] Volgyes D, Pedersen M, Stray-Pedersen A, Waaler D, Martinsen AC. How Different Iterative and Filtered Back Projection Kernels Affect Computed Tomography Numbers and Low Contrast Detectability. J Comput Assist Tomogr. 2017; 41(1):75–81. Epub 2016/08/17. 10.1097/RCT.0000000000000491 . 27529681

